# Correlations among peripapillary vasculature, macular superficial capillaries, and eye structure in healthy and myopic eyes of Chinese young adults (STROBE)

**DOI:** 10.1097/MD.0000000000022171

**Published:** 2020-09-11

**Authors:** Yuxia Guo, Yunlei Pang, YanJie Kang, Xiaoguang Zhang, Han Zhang, Guisen Zhang, Lei Liu

**Affiliations:** a Department of ophthalmology, Baotou Chaoju Eye Hospital, Baotou; b Department of ophthalmology, Chifeng Chaoju Eye Hospital, Chifeng; c Department of ophthalmology, Hohhot Chaoju Eye Hospital, Hohhot; d Department of ophthalmology, First Affiliated Hospital, China Medical University, Shenyang, China.

**Keywords:** anterior chamber depth, circumference of foveal avascular zone, macular vascular density, myopia, peripapillary vascular density

## Abstract

The correlations between retinal vessel distribution, anterior chamber depth (ACD) and other myopic eye structural parameters remains elusive. This study aims to investigate retinal vasculature and eye structure correlations in healthy and myopic eyes of Chinese young adults.

In this cross-sectional study, 181 eyes (97 adults) were recruited. Macular and peripapillary vasculature was quantified by optical coherence tomography angiography. Correlations between retinal vasculature and eye structure were analyzed using multivariable linear regression.

There were significant differences in ACD, spherical equivalent, axial length (AL), superficial macular vascular density (MVD), peripapillary vascular density (PVD) and circumference of foveal avascular zone (FAZ) among emmetropia, low-myopia, moderate-myopia, and high-myopia groups (both *P* < 0.05). Furthermore, ACD had significant positive correlation with AL and FAZ, but negative correlation with PVD. MVD also had a negative correlation with AL (beta = –0.247, *P* < .001). In addition, there was a significant negative correlation between circumference of the FAZ and spherical equivalent as well as central subfield thickness (beta = –0.20, *P* = .005; beta = –0.334, *P* < .001, respectively).

The degree of myopia affected ACD, MVD, PVD, and circumference of the FAZ in eyes of young healthy adults. Meanwhile, ACD has a positive, while retinal vascular system measurements have a negative correlation with increasing severity of myopia.

## 1 Introduction

Currently, myopia has become a common ocular disorder in Asian population.^[1]^ The prevalence of myopia is around 80% or more among young adults in East Asian countries.^[2–4]^ As a major cause of visual impairments, high myopia may lead to many complications such as retinoschisis, chorioretinal atrophy, myopic choroidal neovascularization, and other vision-threatening conditions.^[1]^ Hence, it is very important to investigate the mechanisms to prevent myopia.

Previously, there were some reports regarding morphological changes of vasculature system in eyes with myopia.^[5,6]^ However, due to limitations in imaging modality, it is very difficult to quantify the vasculature system accurately such as using fluorescein angiography or Doppler imaging. With the development of technology, optical coherence tomography angiography (OCTA) makes it easy to conduct quantitative measurements of retinal vasculature system. Vasculature system including superficial macular vascular density (MVD), peripapillary vascular density (PVD), the area (measurement in mm^2^), and circumference (measurement in mm) of the foveal avascular zone (FAZ) could be evaluated by OCTA with split-spectrum amplitude-decorrelation angiography and quantified by integrated software. To date, an increasing number of studies have focused on the morphological changes of the retina vasculature system in eyes with myopia.^[7–13]^ However, the significant sectional correlations between retinal vessel distribution (superficial parafoveal and peripapillary area), anterior chamber depth (ACD) and other myopic eye structural parameters in many works remains elusive, since the previous OCTA studies regarding myopia mainly focused on the association between myopia and vascular structure in macular or peripapillary area, respectively. In addition, the reports on whether their correlations rule out physiological and biochemical factors are limited.

Bearing this in mind, we aimed to perform a cross-sectional study to investigate the comprehensive correlations between retinal vessel distribution via OCTA assessment and structural parameters in healthy and myopic eyes of Chinese young adults.

## 2 Materials and methods

### 2.1 Participants

This cross-sectional study was performed in the Hohhot Chaoju Eye Hospital, Inner Mongolia. The study population consisted of healthy participants with or without myopia who were recruited from October 2018 to December 2018.

### 2.2 Inclusion and exclusion criteria

Inclusion criteria were as follows:

(1) Between the age of 18 and 40 years old;
(2) Eyes with best corrected visual acuity (BCVA) equal to or better than 20/20, spherical equivalent (SE) less than or equal to 0.50 diopter (D), and normal intraocular pressure (IOP).
(3) For each participant, 1 or both eyes meeting the criteria were included in the study. The included eyes were divided into 4 groups according to refraction: emmetropia (EM; mean spherical equivalent (MSE) 0.50D to − 0.50 diopters (D)), low myopia (LM; MSE − 0.75D to − 2.75 D), moderate myopia (MM; MSE − 3.00D to − 5.75 D), and high myopia (HM; MSE less than or equal to − 6.00 D).

Exclusion criteria were:

(1) any history of prior refractive, lens, vitreous, or retinal surgery, or evidence of any retinal disease such as epiretinal membrane, foveoschisis, macular holes, choroidal neovascularization, retinal detachment, neuro-ophthalmic disease, or IOP > 21 mm Hg;
(2) poor-quality OCTA scan images with a signal strength less than 55;
(3) having systemic diseases such as hypertension or diabetes mellitus.

### 2.3 Ophthalmologic and systematic examination

Demographic information, systematic and ophthalmologic medical history were recorded for all participants. Furthermore, all participants underwent a comprehensive ophthalmologic examination including refractive error examination (AR-310A; Nidek, Japan), BCVA, IOP measurement (CT-800 tonometer; Topcon, Japan), slit-lamp biomicroscopy, fundus examination, ACD, and axial length (AL) measurement (Lenstar LS900 Haag-Streit AG, Switzerland). OCTA (Optovue RTVue XR Avanti, Optovue Inc., Fremont, CA) was used for retinal vascularization, central macular thickness (CMT) and retinal nerve fibre layer (RNFL) assessment. This OCTA system has an A-scan rate of 70 kHz scans per second, with a light source centered on 840 nm and a bandwidth of 45 nm. A 6 mm × 6 mm (36 mm^2^) area OCTA acquisition centered on the optic disc was performed, to record the overall RNFL thickness and vessel density. For macular central thickness and superficial vessel density, a 3 mm × 3 mm (9 mm^2^) scanning area centered on the macula was acquired. Furthermore, the area and circumference of the FAZ were also calculated automatically. Two well-trained ophthalmologists examined and assessed all subjects independently. Poor quality image which was with a signal strength index less than 40 was excluded.

On the same day of the OCTA imaging, height, and weight were measured, and body mass index (BMI) was calculated using the following formula: BMI = weight (kg)/height (m^2^). Fasting (more than 8 hour) venous blood samples were obtained to measure for fasting plasma glucose, triglycerides, and total cholesterol levels.

This study was fully approved by the ethics committee of the Hohhot Chaoju Eye Hospital, Inner Mongolia. Informed consent was obtained from each participant.

### 2.4 Statistics

The data were processed and analyzed with statistical analysis software (SPSS for Windows, version 20.0; IBM-SPSS, Chicago, IL). Descriptive statistics (the number and percentage of categorical variables and the mean ± standard deviation of continuous variables) were evaluated in order to determine baseline characteristics. Further, the Wilk–Shapiro test was conducted to explore the distribution of the continuous variables. One-way analysis of variance was used for comparisons of variables among the 4 including eye groups. To compare the categorical data, the Chi-squared test was used. To assess the relationship between the parameters of the macular microvasculature and the structural profiles, Pearson's correlations were used. Furthermore, the relationship between retinal vessel distribution (both macular and peripapillary area), and ACD with other variables was analyzed through univariate linear regression. Finally, age, gender, and any variable with probability *P* less than .05 in univariate linear regression were included in multivariate regression analysis. A two-tail *P* value of less than .05 was considered statistically significant for all analyses.

## 3 Results

There were 181 eyes (97 adults) without pathological changes recruited in current study and divided into 4 groups: emmetropia (n = 44), low myopia (n = 65), moderate myopia (n = 37), and high myopia (n = 35). Thirteen eyes were excluded due to poor fixation and inferior-quality images, and distributed as follow: emmetropia (n = 4), low myopia (n = 7), moderate myopia (n = 1), and high myopia (n = 1). Demographic characteristics, topical and systemic measurements are shown in Table 1. There were statistically significant differences in terms of gender (*P* = .002), SE (*P* < .001), AL (*P* < .001), and ACD measurements (*P* = .004). However, there were no significant differences in age, laterality, IOP, BMI, fasting plasma glucose, triglycerides, total cholesterol, lens thickness, CMT, FAZ and RNFL thickness between the 4 groups (all *P* > .05). For retinal vascular system, there were statistically significant differences in terms of MVD (*P* = .037), PVD (*P* = .034) and the circumference of the FAZ (*P* < .001).

**Table 1:**
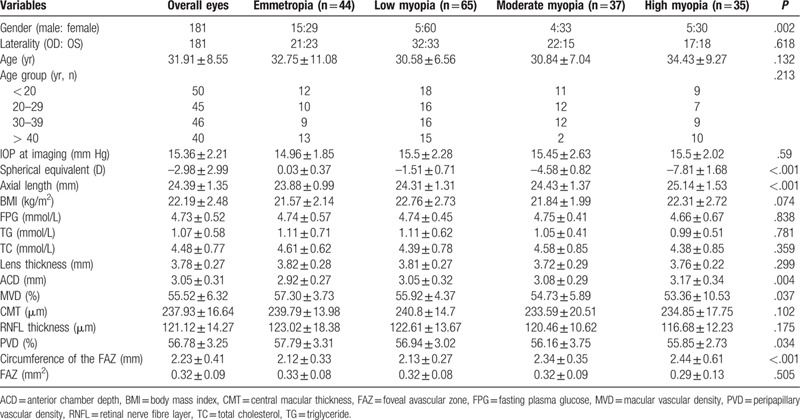
Demographic characteristics among the different myopia groups (by eyes).

**Figure 1:**
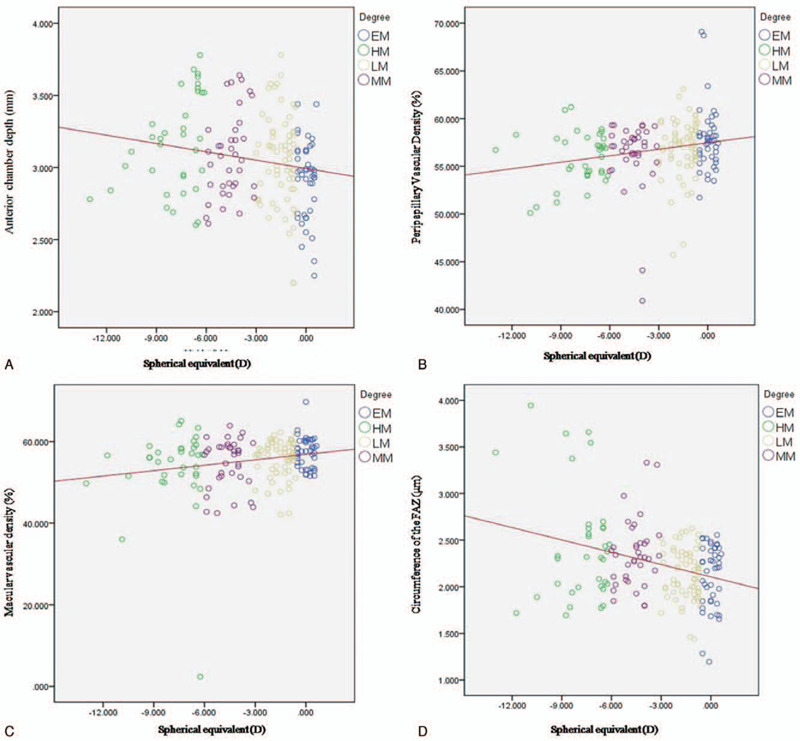
Scatter plots illustrating the linear (red line) associations between spherical equivalent (D) and OCTA macular, peripapillary vascular density, central subfoveal thickness (μm), and anterior chamber depth (mm) measurement of the studied eyes. EM, emmetropia, SE 0.50D to − 0.50 D; LM, low myopia, SE −0.75D to −2.75 D; MM, moderate myopia, SE −3.00D to −5.75 D; HM, high myopia, SE≤−6.00 D.

**Figure 2:**
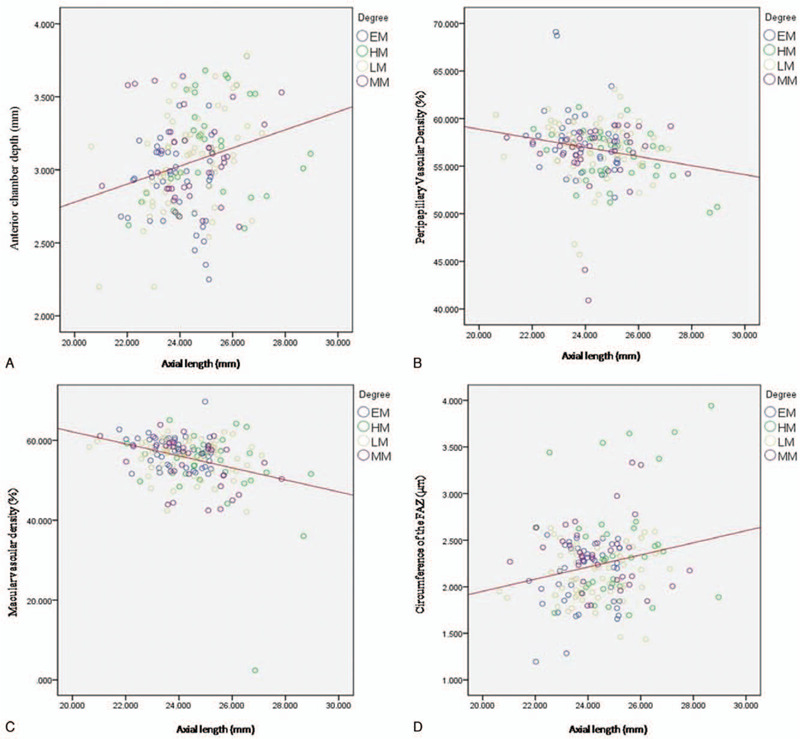
Scatter plots illustrating the linear (red line) associations between axial length (mm) and OCTA macular, peripapillary vascular density, central subfoveal thickness (μm), and anterior chamber depth (mm) measurement of the studied eyes. EM, emmetropia, SE 0.50D to −0.50 D; LM, low myopia, SE −0.75D to −2.75 D; MM, moderate myopia, SE −3.00D to −5.75 D; HM, high myopia, SE≤−6.00.

Furthermore, we looked at the correlation between retinal vascular system, ACD with SE and AL (Table 2). Generally, SE had significantly negative correlation with ACD and circumference of the FAZ, while positive correlation with MVD and PVD (Fig. 1). There was a significant positive correlation between AL and ACD as well as circumference of the FAZ, which appositive with the trend between AL and MVD as well as PVD (Fig. 2).

**Table 2:**
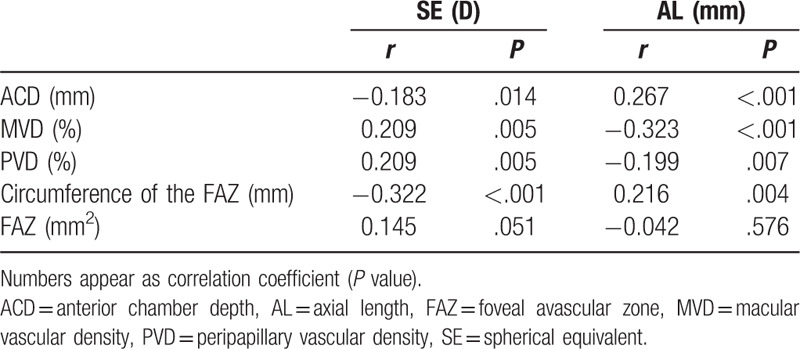
Correlation between overall and regional peripapillary vessel density and myopic measurements.

In the results from univariate linear regression analysis (Table 3), we found that ACD was associated SE (*P* = .014), AL (*P* < .001), PVD (*P* = .002) and FAZ (*P* = .042). MVD was associated with SE (*P* = .005), AL (*P* < .001) and circumference of the FAZ (*P* = .007). Further, PVD was also associated with SE (*P* = .005) and AL (*P* = .007). Moreover, circumference of the FAZ was associated with gender (*P* = .031), SE (*P* < .001), AL (*P* = .004) and CMT (*P* < .001), and MVD (*P* = .007).

**Table 3:**
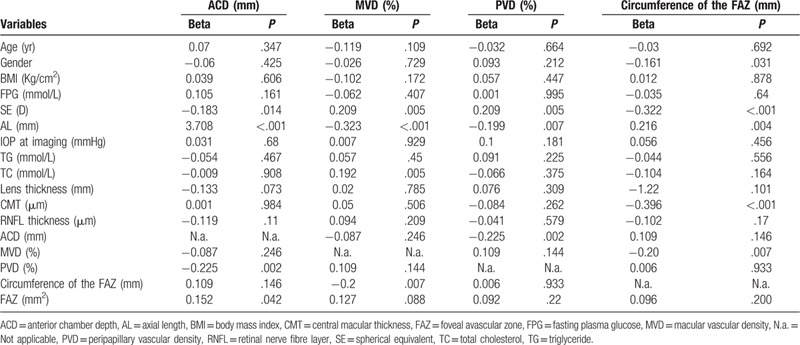
Univariate linear regression analysis of factors affecting the average anterior chamber depth, macular vascular density, peripapillary vessel density, circumference of the FAZ.

Taken together that there was a significant difference on gender among 4 groups, we adjusted age and gender with any variable which was significant by univariate linear regression to investigate the correlation factors for ACD, MVD, PVD, and circumference of the FAZ (Table 4). We found that ACD had significant positive correlation with AL (beta = 0.218, *P* = .004) but negative correlation with PVD (beta = –0.156, *P* = .003). There was a significant inverse correlation between MVD and AL (beta = –0.247, *P* < .001). Furthermore, there was a significant negative correlation with circumference of the FAZ with SE as well as CMT (beta = –0.20, *P* = .005; beta = –0.334, *P* < .001, respectively).

**Table 4:**
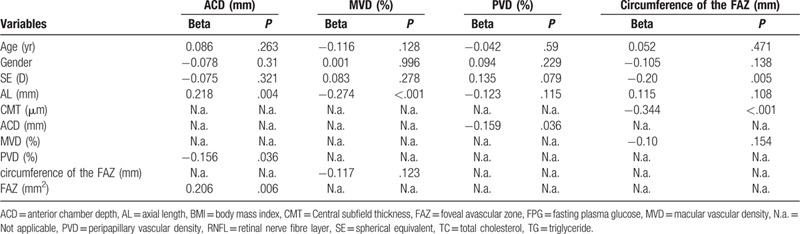
Multivariate linear regression analysis of factors affecting the average anterior chamber depth, macular vascular density, peripapillary vessel density, circumference of the FAZ.

## 4 Discussion

In this cross-sectional observation study, we performed a quantitative assessment of retinal vasculature system using OCTA and compared these measurements as well as eye structure measurements among Chinese adults with and without myopia considering biometric factors. Our findings suggested that there were significant differences on SE, AL, ACD, MVD, PVD, and circumference of the FAZ among low-, moderate-, high-myopic and emmetropia eyes. Both SE and AL had significant associations with ACD, MVD, PVD, and circumference of the FAZ, respectively. After multivariable regression, ACD had a significant positive correlation with AL and FAZ but a negative correlation with PVD. In addition, AL, as an independent factor, was negatively correlated with MVD. Furthermore, circumference of the FAZ had negative correlation with SE as well as CMT. These relationships are crucial in understanding changes to the myopic eye, which is potentially progressing to degenerative myopia.

Our findings provided some evidence that ACD measurements could be affected by the degree of myopia, and this was supported by the Saudi, Iranian and Chinese studies which found ACD to be increased between emmetropes and myopes.^[14–16]^ However, another study in Malaysian adult population found that ACD could not be affected by the severity of myopia (i.e., low, moderate, and high).^[17]^ The inconsistent conclusions between the present and previous studies could be due to ethnicity.^[18,19]^

Currently, OCTA with split-spectrum amplitude-decorrelation angiography can provide noninvasive and quantitative retinal vascular information in detail. Regional retinal vascular measurements such as MVD, PVD as well as circumference of the FAZ, can be calculated in an OCTA image. In this study, we used OCTA to quantitatively evaluate these measurements and found significant differences among emmetropia, low myopia, moderate myopia, and high myopia. We found that there were significant associations between ACD, MVD, PVD, circumference of the FAZ and SE as well as AL. Furthermore, the correlations among those measurements were various by multivariable linear regression.

Generally, among all including eyes (myopia and emmetropia), ACD was positively associated with AL while negative associated with PVD. Our finding was highly consistent with a prior study which revealed that the severity of myopia could affect ACD measurements. In the myopic eye, the eye becomes more elongated because of increasing AL, which may lead to an increase in ACD.^[15]^ Moreover, we found that high myopic eyes had lower PVD which was consistent with previous findings.^[20–22]^ Interestingly, PVD was independently associated with ACD; nonetheless, no report has shown a relationship between PVD and ACD in cases of myopia. Thus, further studies focusing on correlation between ACD and PVD among myopic individuals may be needed.

For macular retinal vascular measurement, our results showed that MVD was lower in the high myopia group than in other groups. Similarly, a prior study found a significant decrease in MVD with increasing level of myopia severity.^[12]^ In contrast, Yang et al found that varying severities of myopia did not affect MVD in Chinese young healthy adults.^[11]^ In the study by Yang and associates, only myopic eyes without pathological changes were recruited. The study by Mo et al revealed that macular flow density decreased in pathological myopia compared to those in high myopia and emmetropia.^[21]^ Considering that no effect on MVD is prominent in myopic eyes without pathological changes, particularly in young subjects, we included eyes with or without pathological changes but with BCVA equal to or better than 20/20 to avoid the effects of the pathological changes. We demonstrated that more severe myopia was associated with decreased MVD in young myopic eyes with or without pathologic changes. Furthermore, another study using OCTA assessment revealed that both superficial and deep MVD were associated with AL but not considered with potential variables.^[12]^ In our study, multivariable linear regression indicated that there is a negative correlation between MVD and AL that is not mediated by other variables such as age, gender, SE, AL, and circumference of the FAZ.

With OCTA, we can assess the circumference of the FAZ measurement. In current study, we found that circumference of the FAZ was increased with the increasing degree of myopia and independently correlated with SE and CMT. Due to no significant difference on CMT among the 4 groups in our study, it is difficult to deduce the correlation between circumference of the FAZ and CMT as well as myopia. However, we can deduce that with the increasing degree of myopia, the MVD changes together with circumference of the FAZ increasing. The mechanism of decreased blood flow in myopic eyes is still unclear. Our finding suggests that there was a potential link between the eye structural and vascular system changes in the parafoveal area of myopic eyes. Of note, does structural change or vascular change happen first? One possible answer is that if the demand is diminished, the supply is reduced.^[23,24]^ On the other hand, reduced retinal vascular parameters are due to the elongation of the globe. Generally, a longitudinal, larger sample size cohort study using OCTA might be able to improve our knowledge on this.

It should be noted that there were some limitations in current study. First, our study was of observational and cross-sectional design, relatively small sample size and from the same race within a small range of age, thus, its conclusions should be concerned with cautious. Secondly, we did not divide the macular and peripapillary retinal vessel density into different regional layers or quadrants. Thirdly, our quantitative retinal vascular system was not confirmed by another assessment method, thus, our conclusions may not be applicable to the other techniques such as Doppler or fluorescence angiography. Further longitudinal studies with big sample size and more detailed division are required to investigate the vascular and structural changes in the myopic eyes.

## 5 Conclusion

In conclusion, according to OCTA, we found that ACD increased significantly as the AL increased, while there was a negative correlation between ACD and PVD in the myopic eyes. Further, MVD and circumference of the FAZ decreased significantly as the degree of myopia increased. Although there is no difference on CMT measurement between emmetropic and myopic eyes, circumference of the FAZ has a reverse correlation with CMT. Generally, measuring changes in retinal vascular system with OCTA and eye structure could be a useful guide to assess the pathology and mechanism of disease in myopic individuals.

## Author contributions

Conception and design of the study: GSZ and LL.

Data acquisition: YXG, YLP, YJK and LL.

Data management and analysis: YJK and XGZ

Manuscript drafting/editing: GSZ, HZ and LL.

All authors read and approved the final manuscript.
